# Investigation of the metabolic stability of olmutinib by validated LC-MS/MS: quantification in human plasma

**DOI:** 10.1039/c8ra08161a

**Published:** 2018-12-04

**Authors:** Mohamed W. Attwa, Adnan A. Kadi, Hany W. Darwish, Ali S. Abdelhameed

**Affiliations:** Department of Pharmaceutical Chemistry, College of Pharmacy, King Saud University P. O. Box 2457 Riyadh 11451 Kingdom of Saudi Arabia hdarwish@ksu.edu.sa mzeidan@ksu.edu.sa akadi@ksu.edu.sa asaber@ksu.edu.sa +966 1146 76 220 +966 1146 77343; Analytical Chemistry Department, Faculty of Pharmacy, Cairo University Kasr El-Aini St. Cairo 11562 Egypt

## Abstract

Olmutinib (OTB, Olita™) is an orally available third-generation epidermal growth factor receptor tyrosine kinase inhibitor (EGFR TKI). It was developed by Boehringer Ingelheim and Hanmi Pharmaceutical Co. Ltd for the cure of non-small cell lung cancer (NSCLC). In May 2016, OTB was approved in South Korea for the treatment of patients suffering from metastatic or locally advanced EGFR T790M mutation-positive NSCLC. A LC-MS/MS methodology was validated for OTB quantification in human plasma. An extended application for this validated LC-MS/MS is OTB metabolic stability evaluation. Chromatographic separation of OTB and ponatinib (PNT, IS) was attained using a reversed phase with isocratic elution. The linearity of the developed LC-MS/MS method ranged from 5.00 to 500.00 ng mL^−1^ with *r*^2^ ≥ 0.9999 in human plasma. LOD and LOQ were 1.12 and 3.39 ng mL^−1^, respectively. The intra-day and inter-day precision and accuracy were 1.17 to 2.75% and 97.86 to 101.48%, respectively. The intrinsic clearance (CL_int_) was 2.71 mL min^−1^ kg^−1^ and the *in vitro* half-life (*t*_1/2_) was 48.80 min. A review of the literature revealed that there are no previous articles about the quantification of OTB in human plasma using LC-MS/MS or its metabolic stability assessment.

## Abstract 2

Olmutinib (OTB, Olita™) is an orally available third-generation epidermal growth factor receptor tyrosine kinase inhibitor (EGFR TKI).

## Graphical abstract

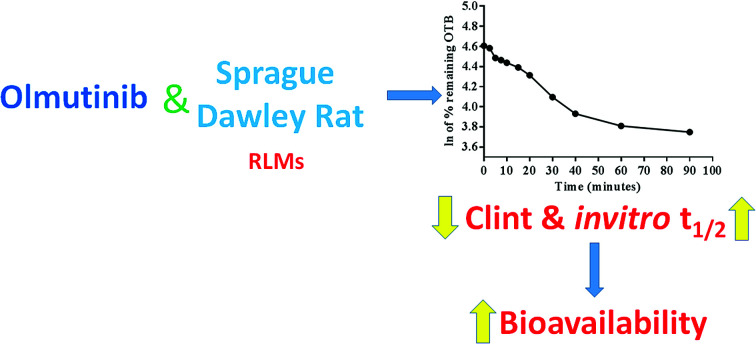

## 1. Introduction

Cancer is considered as one of the major reasons of death, and it causes more than one fourth of the world's deaths.^1^ Molecular targeting strategies have lately been utilized for curing disseminated cancer.^2^ From all types of cancers reported worldwide, lung cancer is the major reason of death because it was responsible for around 20% of cancer deaths (1.59 million deaths) in 2012.^3^ Non-small cell lung cancers (NSCLCs) represent almost 90% of lung cancers.^4,5^ Patients suffering from advanced-stage NSCLCs accompanied by active mutations of epidermal growth factor receptors (EGFR) have received a first-line targeted medication of tyrosine kinase inhibitors (TKIs).^6^ The first-generation TKIs such as gefitinib and erlotinib have a very good initial response against these active mutations of EGFR.^7^ However, a major drawback for this group appears during the first year of treatment, which is the acquired resistance development in most patients.^7,8^ This has led to vigorous attempts by scientists to develop the second generation of EGFR TKIs (*e.g.*, avitinib, dacomitinib, *etc.*), which is irreversibly linked to a domain in EGFR called TK.^8,9^ The third-generation TKIs exhibit the advantages of the second-generation drugs by hindering mutations of EGFR and conquering T790M resistance mutation with very reasonable selectivity for these mutations over other wild-type EGFR.^8,9^

**Fig. 1:**
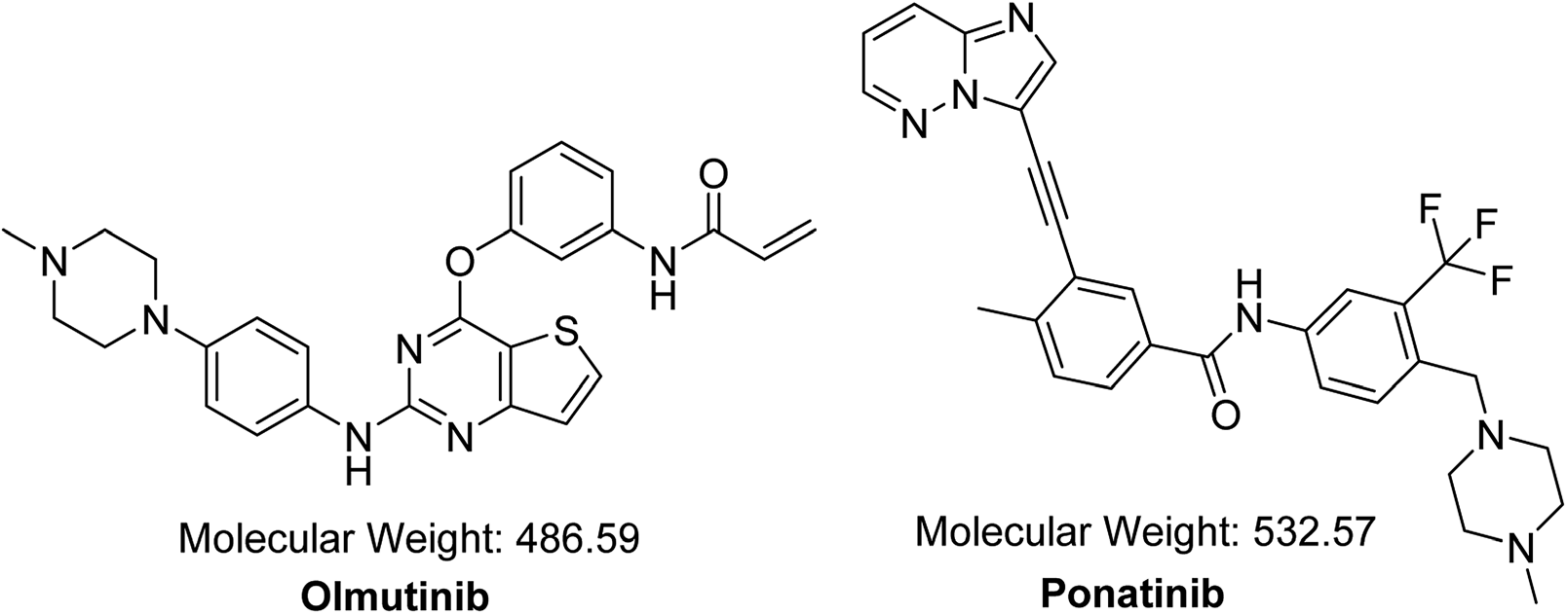
Chemical structures of OTB and PNT (IS).

OTB (HM61713, Fig. 1) is an orally available EGFR TKI. Its IUPAC name is *N*-{3-[(2-{[4-(4-methyl-1-piperazinyl)phenyl]amino}thieno[3,2-d]pyrimidin-4-yl)oxy] phenyl} acrylamide. The FDA granted a breakthrough therapy designation for OTB in NSCLC.^10^ In May 2016, OTB was approved in South Korea under the trade name Olita™ for management of patients suffering from metastatic or local advanced EGFR T790M mutation-positive NSCLC.^10^

A systematic literature review for OTB showed that there is one reported analytical method to quantify OTB in rat plasma, where the percent recovery of OTB in rat plasma was 85.8% to 95.5%.^11^ The reported method is a gradient method with a pharmacokinetic application. These findings motivated us to develop a validated isocratic LC-MS/MS method for estimating OTB with high recovery in human plasma. Our current developed proposed procedure is an isocratic method with very high reproducible recovery (99.61 ± 2.82%, with RSD less than 2.17%) for OTB quantification in human plasma with applications to evaluate OTB metabolic stability. For the evaluation of OTB metabolic stability, two important parameters were computed. These two parameters are intrinsic clearance and *in vitro* half-life (*t*_1/2_) that could be used for calculating *in vivo t*_1/2_, hepatic clearance and bioavailability, which gives an idea about the investigated compound metabolism. If the tested compound is rapidly metabolized in the human body, it can possess low bioavailability.^12^

## 2. Experimental

### 2.1. Reagents and chemicals

All chemicals and reagents are listed in Table 1. Human plasma was kindly supplied by King Khaled University Hospital (Riyadh, KSA) after permissions were obtained from human donors and stored at −70 °C until usage. RLMs were prepared in-house using Sprague Dawley rats.^13–16^ Maintenance of the rats was done following the Animal Care Center guidelines at the College of Pharmacy at King Saud University (KSU) in Saudi Arabia. The animal experiment protocol was approved by the Institutional Review Board at KSU. The Ethics Review Committee at KSU approved the experimental design that was utilized for animal study.

**Table:** List of chemicals and reagents

Name^a^	Source
Olmutinib (OTB) (99.95% pure)	Med Chem. Express (USA)
Ponatinib (PNT) (98.96% pure)	LC Laboratories (USA)
Acetonitrile (ACN) and formic acid (HCOOH)	Sigma-Aldrich (USA)
Water (HPLC grade)	Milli-Q Plus instrument (USA)
RLMs	Prepared in lab using Sprague Dawley rats^13–16^
Human plasma	King Khaled University Hospital (KSA)
Sprague Dawley rats	The Animal Care Center at King Saud University (KSA)

a Reference powders are of analytical quality and all used solvents are HPLC quality.

### 2.2. LC-MS/MS methodology

All liquid chromatographic and mass spectrometric parameters were adjusted to achieve fast separation with high resolution. Table 2 shows all adjusted LC-MS/MS parameters. An isocratic elution mobile phase was used for the separation of OTB and IS.

**Table:** Optimized parameters of the established LC-MS/MS methodology

LC parameters		MS parameters	
RRLC	Agilent 1200	MS	Agilent 6410 Triple Quadrupole
Isocratic mobile phase	40% ACN	Ionization source	Positive ESI
	60.00% aqueous (0.10% formic acid)		Drying gas: low purity N_2_ gas
	Flow rate: 0.25 mL min^−1^		Flow rate at 12.00 L min^−1^
	Injection volume: 5 μL		Pressure at 60.00 psi
Eclipse plus C_18_ column	50 mm in length		Source temperature at 350.00 °C
	2.10 mm ID		Capillary voltage at 4000.00 V
	1.80 μm particle size	Collision cell	Collision gas: high purity N_2_ gas
	*T*: 22.00 ± 1.00 °C	Mode	Multiple reaction monitoring (MRM)
Analyte	OTB	OTB MRM transitions	*m*/*z* 487 → *m*/*z* 432, FV: 110.00 V, CE: 25.00 eV
			*m*/*z* 487 → *m*/*z* 402, FV: 145.00 V, CE: 15.00 eV
IS	PNT	PNT MRM transitions	*m*/*z* 533 → *m*/*z* 433 FV: 135.00 V, CE: 20.00 eV
			*m*/*z* 533 → *m*/*z* 260 FV: 140.00 V, CE: 25.00 eV

PNT was chosen as IS in OTB analysis as the same method of extraction was applied successfully to OTB and PNT (OTB and PNT recoveries were 99.91 ± 1.47% and 98.19 ± 1.10% in human plasma, respectively) and the elution time of PNT was close to that of OTB with good resolution; thus, the method is fast with a short run time. Both PNT and OTB are TKIs and are not prescribed together for patients; thus, this method can be utilized for clinical applications such as pharmacokinetics or therapeutic drug monitoring (TDM) for patients under olmutinib treatment.

**Scheme 1:**
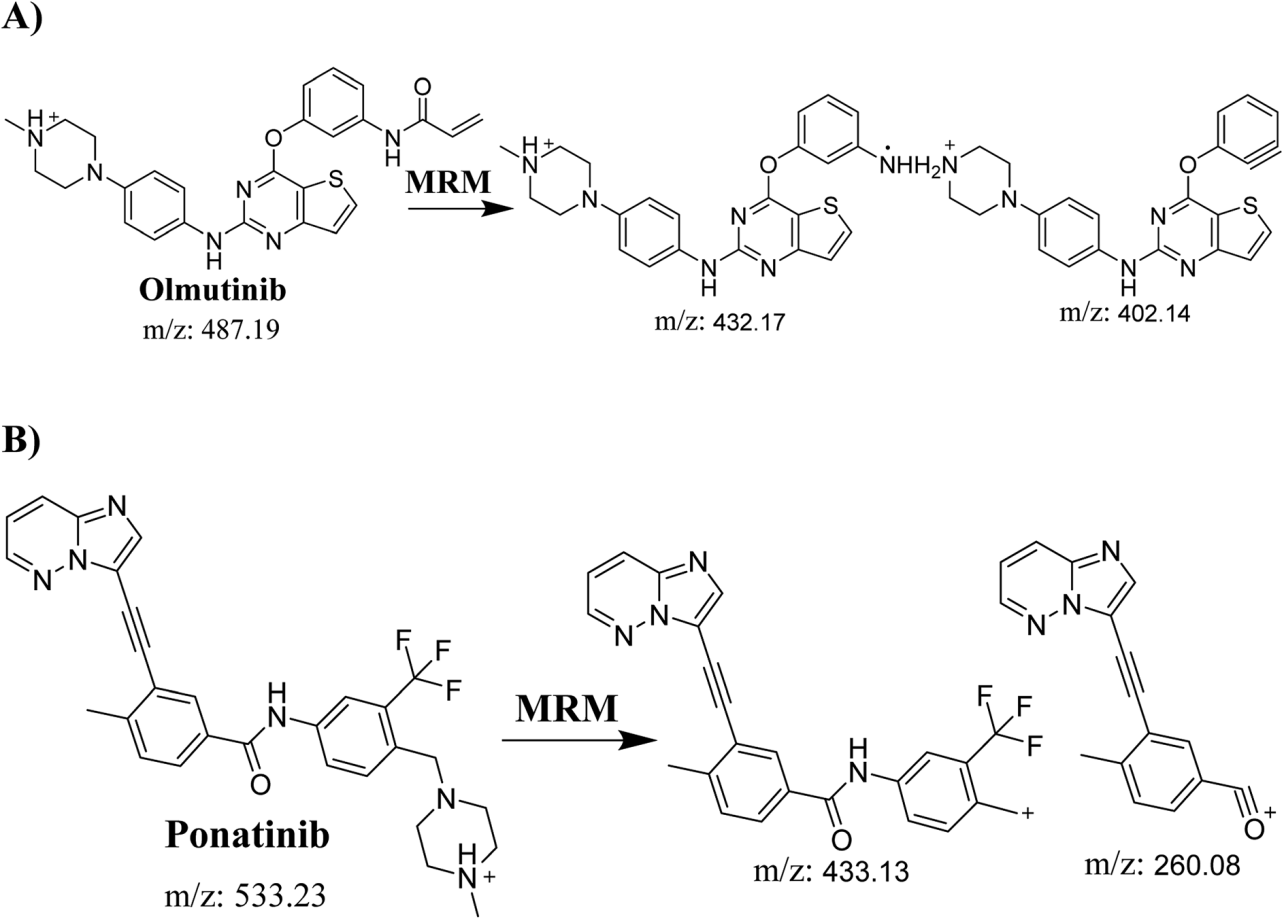
MRM ions of (A) OTB and (B) PNT (IS).

**Fig. 2:**
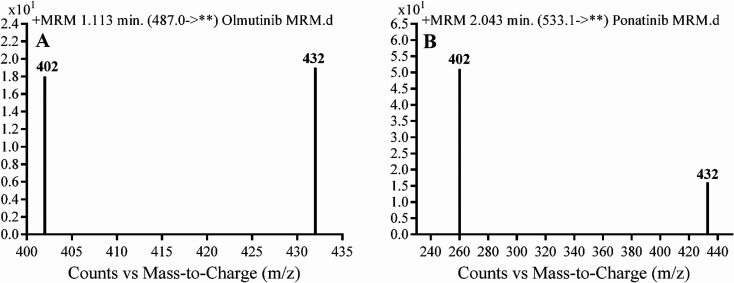
MRM mass transition spectra of (A) olumtinib and (B) PNT (IS).

Detection was conducted on a QqQ mass detector connected to an electrospray ionization source (ESI) that was operated with a positive mode. Low-purity nitrogen (11 L min^−1^) was used as a drying gas in the ESI source and high-purity nitrogen (50 psi) was used as a collision gas inside the collision cell of the mass spectrometer. The ESI temperature and capillary voltage were adjusted to 350 °C and 4000 V, respectively. The Mass Hunter software of Agilent was used to control the instruments and manage the data acquisition. Quantifying OTB was done using the multiple reaction monitoring (MRM) analyzer mode for the mass transitions (parent to product ion) from 487 → 432 and 487 → 402 for OTB and from 533 → 433 and 533 → 260 for PNT (IS) (Scheme 1). The fragmentor voltage (FV) was adjusted to 110 and 145 V with collision energies (CE) of 25 and 15 for OTB (FV of 135 and 140 V with CE of 20 and 25 for PNT). The MRM mode of the mass analyzer was used for OTB quantification to remove any interference from human plasma constituents and elevate the sensitivity of the developed LC-MS/MS methodology (Fig. 2).

### 2.3. Standard solutions of OTB

OTB (1.00 mg mL^−1^) was solubilized in DMSO. Afterwards, the stock solution was diluted ten times with the mobile phase to prepare working solution 1 (100.00 μg mL^−1^) that was further diluted ten times with the mobile phase to prepare working solution 2 (10.00 μg mL^−1^). PNT stock solution (IS, 100.00 μg mL^−1^) was solubilized in DMSO. A working solution of PNT was made by diluting stock of PNT (IS) 50.00 times with the mobile phase to prepare working solution 3 (2.00 μg mL^−1^).

### 2.4. Preparation of OTB calibration standards

OTB working solution 2 (10.00 μg mL^−1^) was mixed with certain volumes of human plasma matrix to generate fourteen calibration points: 5.00, 10.00, 15.00, 20.00, 30.00, 40.00, 50.00, 80.00, 100.00, 150.00, 200.00, 300.00, 400.00 and 500.00 ng mL^−1^. Three calibration standards 15.00 ng mL^−1^, 150.00 ng mL^−1^ and 400.00 ng mL^−1^ were selected as quality controls: low (LQC), medium (MQC) and high (HQC), respectively. Extraction was done using a protein precipitation technique by ACN. First, alkalinization of spiked plasma standards was conducted by adding 1 mL of NaOH/glycine buffer (0.10 M, pH 9.5) followed by mixing for 30 seconds using a vortex. Second, two mL of ACN was added, followed by centrifugation at 14 000 rpm (12 min at 4 °C) to aid in protein precipitation.^17^ Supernatants were collected and then, filtration was done using a syringe filter (0.22 μm pore size). Fifty μL of PNT working solution 3 was added to 1 mL of the filtered samples and transferred to HPLC vials to be loaded into LC-MS/MS for analysis. Similarly, blank samples were prepared by utilizing the stated mobile phase in place of plasma to verify the absence of any interference from plasma components at RT of OTB and IS. A calibration curve was constructed for spiked human plasma by plotting the peak area ratio of OTB to PNT (*y* axis) against OTB standard nominal values (*x* axis). A linear regression equation was used to express the linearity of the established method. The slope, *r*^2^ and intercept values were calculated.

### 2.5. Method validation

The validation parameters of the LC-MS/MS methodology that was developed to estimate OTB in human plasma were mentioned in detail earlier.^17,18^ They were calculated based on the assay recovery, linearity, sensitivity, specificity, reproducibility, limit of quantification (LOQ) and limit of detection (LOD). All these parameters were computed for OTB.

### 2.6. Metabolic stability of OTB

The metabolic stability of OTB was studied by measuring the decrease in OTB concentration when it was incubated with RLMs. One μM OTB was incubated with 1.00 mg mL^−1^ microsomal proteins in triplicate. All incubations were kept for 10 minutes to reach 37 °C in a water bath. Initiation of the metabolic pathway was performed by adding 1 mM NADPH in phosphate buffer (pH 7.4) that contained 3.30 mM MgCl_2_. Two mL ACN was added to terminate the metabolic pathway at certain time points: 0.00, 2.50, 5.00, 7.50, 10.00, 15.00, 20.00, 40.00, 60.00, 90.00 and 120.00 min. The same method of extraction as mentioned above was utilized for OTB extraction. The metabolic stability curve for OTB was constructed.

## 3. Results and discussions

### 3.1. HPLC-MS/MS methodology

Optimizations of the chromatographic parameters including pH, mobile phase constituents and RRLC column were conducted. The pH of the aqueous part (0.1% formic acid) was adjusted to 3.2 as at pH values higher than this value, increase in elution time and peak tailing were observed. The ratio of mobile phase components (aqueous phase to ACN) was adjusted to 60% : 40% as more ACN led to overlapping of peaks with poor resolution and less ACN led to a delayed elution time. Various columns (Hilic columns) were checked but OTB and PNT were not retained. The best outcomes were obtained using a C18 column.

**Fig. 3:**
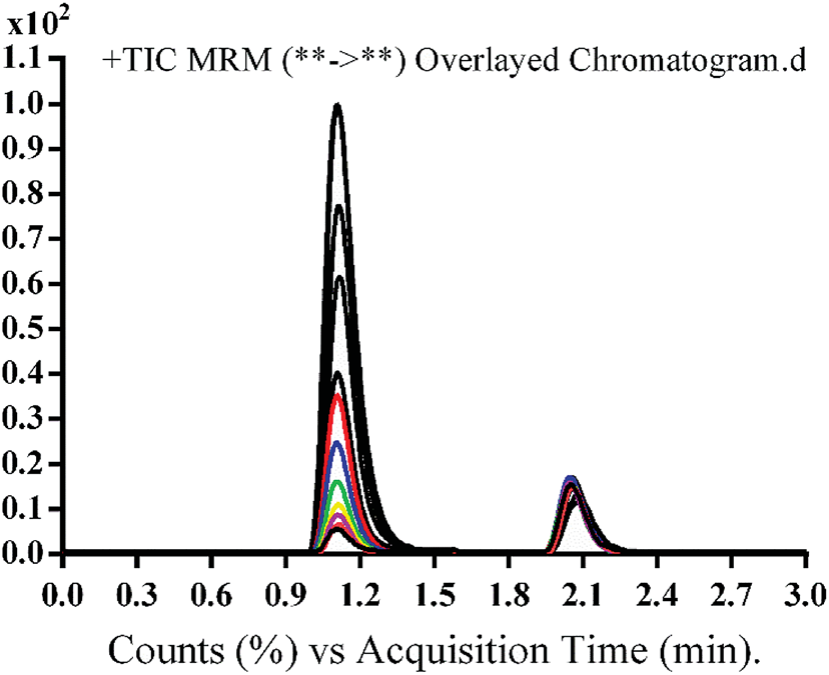
MRM TIC of OTB calibration standards (5–500 ng mL^−1^) and PNT (50 ng mL^−1^).

The chromatographic separation of OTB and PNT (IS) was attained in 3 min. OTB and PNT chromatographic peaks resolved well in the absence of carryover in the blank samples: either the plasma matrix sample or plasma plus internal standard. Fig. 3 shows overlaid MRM total ion chromatograms (TIC) of OTB calibration standards.

### 3.2. Method validation of the developed LC-MS/MS method

#### 3.2.1. Specificity

**Fig. 4:**
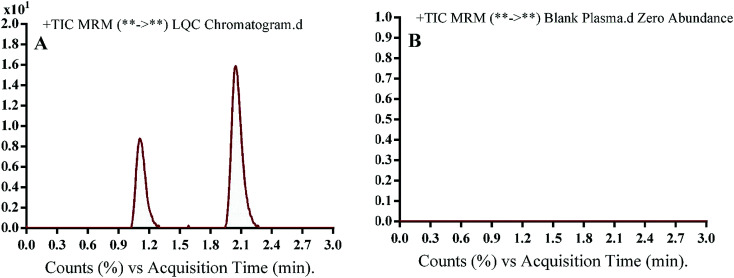
MRM TIC of OTB lower quality control sample in human plasma (A) and blank human plasma (B) showing no matrix interference.

Fig. 4 shows excellent separation of OTB and PNT peaks and lack of any peak in the blank plasma matrix at their corresponding elution times, which ensured the developed methodology specificity. No carryover effect of OTB and PNT was seen in the MS chromatograms.

#### 3.2.2. Sensitivity and linearity

The least square statistical method was utilized to compute calibration curve equations (*y* = *ax* + *b*). A linear fit was verified by the coefficient of determination (*r*^2^), which was linear in the range from 5 ng mL^−1^ to 500 ng mL^−1^. The recovery for OTB detected in the spiked human plasma samples was 99.61 ± 2.82% with RSD less than 2.17%.

The linearity range and correlation coefficient (*r*^2^) for the supposed methodology were 5–500 ng mL^−1^ and ≥0.9999, respectively, in human plasma. The calibration curve regression equation of OTB in human plasma was *y* = 3.69*x* − 5.67. LOD and LOQ were 1.12 and 3.39 ng mL^−1^, respectively, in human plasma.

The RSD values of six repetitions for each concentration point in the calibration curve were lower than 3.63% in human plasma. Back calculation for the fourteen samples of OTB in plasma (calibration standards and QC levels) confirmed the performance of the developed methodology. The reproducibility and repeatability of the method were represented by the intra-day and inter-day accuracy and precision, which were 1.17 to 2.75% and 97.86 to 101.48%, respectively (Table 3). The mean percentage of OTB recoveries was 99.61 ± 2.82% in the human plasma matrix.

**Table:** Data of OTB back-calculated concentrations of the calibration levels from the human plasma matrix

Nominal Concentrations of OTB in ng mL^−1^	Mean^a^	SD	RSD%	Accuracy%
5.00	5.10	0.14	2.73	101.98
10.00	10.10	0.24	2.36	101.04
15.00	15.27	0.45	2.93	101.83
20.00	19.72	0.56	2.85	98.59
30.00	28.76	0.61	2.13	95.88
40.00	39.22	1.42	3.63	98.04
50.00	49.59	0.85	1.71	99.19
100.00	96.29	1.50	1.56	96.29
150.00	147.91	3.33	2.25	98.61
200.00	196.49	3.06	1.56	98.25
300.00	305.67	3.96	1.29	101.89
400.00	392.84	7.78	1.98	98.21
500.00	494.15	4.90	0.99	98.83

a Average of six replicates.

#### 3.2.3. Precision and accuracy

As mentioned in Table 4, the intra- and inter-day accuracy and precision values are accepted according to the US FDA guidelines.^19^

**Table:** Precision and accuracy (intra-day and inter-day) of the developed methods

Human plasma	LQC, 15.00 ng mL^−1^		MQC, 150.00 ng mL^−1^		HQC, 400.00 ng mL^−1^	
	Intra-day^a^	Inter-day^b^	Intra-day	Inter-day	Intra-day	Inter-day
Mean	15.22	15.07	146.78	147.26	392.85	393.52
SD	0.42	0.37	2.18	2.47	5.57	4.59
Precision as % RSD	2.75	2.47	1.49	1.68	1.42	1.17
% accuracy	101.48	100.47	97.86	98.17	98.21	98.38

a Average of twelve replicates from day 1.

b Average of six replicates from three consecutive days.

#### 3.2.4. Matrix effects and extraction recovery

Table 5 depicts the recovery percentages of QCs for assessing the OTB concentration in human plasma. OTB and PNT recoveries were 99.91 ± 1.47% and 98.19 ± 1.10% in human plasma, respectively. The absence of the matrix effect on OTB or PNT (IS) was verified by analyzing six different batches of plasma, in which these batches were extracted and spiked with OTB LQC (15 ng mL^−1^) and PNT (50 ng mL^−1^). The above-mentioned batches were labeled as set 1. Preparation of set 2 was conducted using a similar method, but the mobile phase was used instead of human plasma. Thus, the matrix effect was calculated utilizing the following equation:
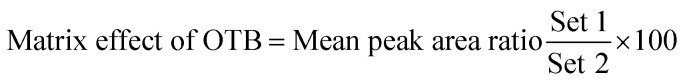

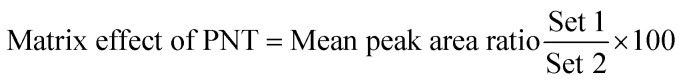


Recovery of OTB QC samples in human plasma matrix

**Table:** 

Quality controls	Human plasma matrix		
	LQC, 15.00 ng mL^−1^	MQC, 150.00 ng mL^−1^	HQC, 400.00 ng mL^−1^
Mean^a^	15.43	146.85	392.29
Recovery (%)	102.87	97.90	98.07
SD	0.34	2.29	6.10
Precision (RSD%)	2.17	1.56	1.56

a Average of six replicates.

**Table:** 

IS conc. (50 ng mL^−1^)	Mean	Recovery%	SD
PNT (IS)	49.09	98.19	1.10

The studied human plasma that contains OTB and PNT showed values of 98.4 ± 2.5% and 98.3 ± 1.54%, respectively. The internal standard normalized matrix effect (IS normalized MF) was computed from the following equation:
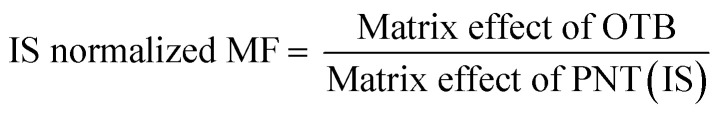


The IS normalized MF is 1.0 and it lies within the acceptable range.^20^ Accordingly, these results proved that the human plasma matrix has no noticeable effect on the ionization of OTB and PNT (IS).

#### 3.2.5. Stability

QC samples were used for stability studies of OTB under many conditions. Table 6 shows the outcomes of such studies, where SD of the mean is lower than 5.10%. Additionally, no observed change of OTB occurred throughout sample storage and handling under the inspected conditions. Also, the results showed the stability of plasma samples containing OTB under normal laboratory conditions with no remarkable loss of OTB concentration.

**Table:** OTB stability in human plasma matrix under various laboratory conditions

Quality controls^a^	Mean ± SD	% of recovery	Precision (RSD%)
**Room temp. for 8h**			
15.00	14.76 ± 0.22	98.40	1.48
150.00	147.26 ± 2.47	98.17	1.68
400.00	393.52 ± 4.59	98.38	1.17

**Three freeze–thaw cycles**			
15.00	14.84 ± 0.42	98.95	2.80
150.00	146.70 ± 5.00	97.80	3.41
400.00	399.19 ± 6.25	99.80	1.57

**24 h storage at 4** ^**o**^ **C**			
15.00	14.74 ± 0.50	98.23	3.40
150.00	145.07 ± 3.79	96.71	2.61
400	390.14 ± 5.50	97.53	1.41

**30 days storage at −20** ^o^C			
15.00	14.33 ± 0.36	95.52	2.55
150.00	144.15 ± 4.03	96.10	2.80
400.00	388.49 ± 4.92	97.12	1.27

a Conc. in ng mL^−1^.

### 3.3. Metabolic stability

**Fig. 5:**
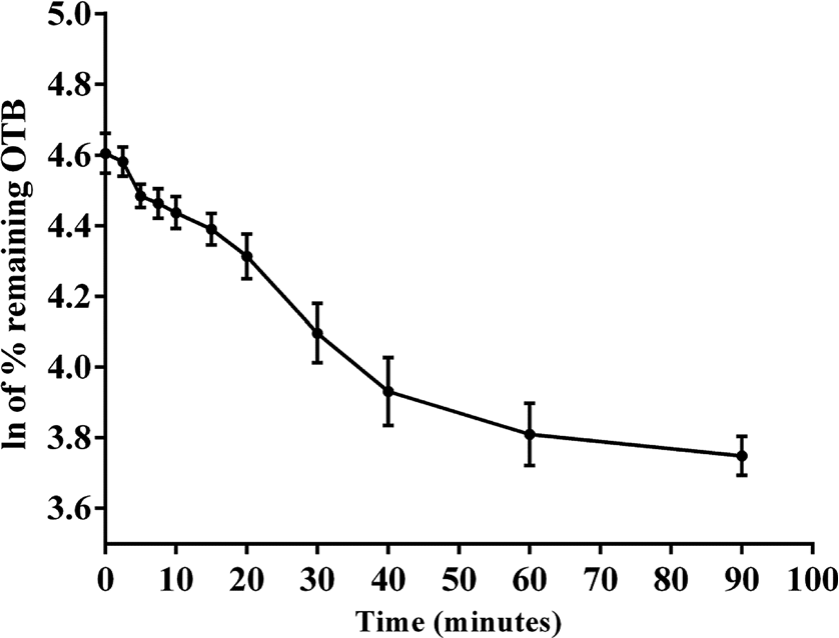
OTB metabolic stability curve.


The metabolic stability of OTB in the RLM preparation was computed. The metabolic pathway was initiated using NADPH as a cofactor and terminated using ACN for enzyme deproteination. Stopping of the metabolic pathway occurred at various time points and extraction of OTB from different incubation points was performed following the same extraction procedure. The OTB concentration in the RLM matrix was computed by the displacement of the peak area ratios in the calibration curve regression equation. The experiment was repeated three times to confirm the results. The metabolic stability curve was drawn by plotting ln(percent of OTB remaining) on the *y*-axis against time of incubation on the *x*-axis (Fig. 5). The linear part regression equation of the plotted curve was used for *in vitro t*_1/2_ calculation^21^ using the following equations:

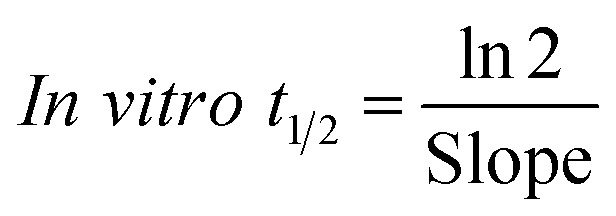


The slope was 0.0142.
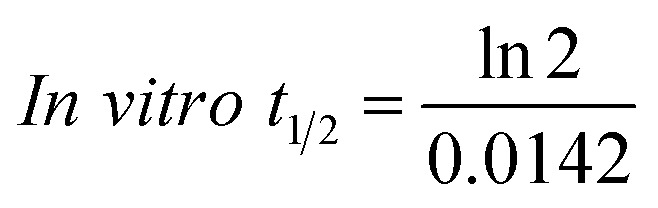
*In vitro t*_1/2_ = 48.8 min.

The intrinsic clearance of OTB was calculated following the *in vitro t*_1/2_ method^12^ using the following equation:
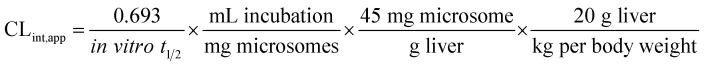

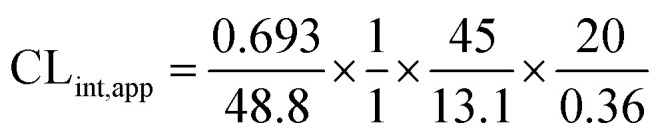
CL_int,app_ = 2.71 mL min^−1^ kg^−1^

From the previous results, the metabolic assessment of OTB was characterized by a lower value of CL_int_ (2.71 mL min^−1^ kg^−1^) and a longer *in vitro t*_1/2_ value (48.8 min), which resulted in slower clearance of OTB from the blood by the liver and probable high *in vivo* bioavailability (Table 7).

**Table:** Metabolic stability parameters of OTB incubation with RLMs for specific time intervals

OTB metabolic stability parameters				
Time (min.)	Conc. (ng mL^−1^)	*X* a	Parameter	Value
0	473.00	4.61	Regression equation^b^	*y* = −0.0142*x* + 4.5894
2.50	461.96	4.58		
5.00	429.24	4.48	*R* ^2^ c	0.9836
7.50	463.01	4.46		
10.00	460.71	4.44	Slope	0.0142
15.00	451.49	4.39		
20.00	437.91	4.31	*t*_1/2_ d	48.80 min
40.00	380.55	4.10		
60.00	401.23	3.93	CL_int_^e^	2.71 mL min^−1^ kg^−1^
90.00	418.84	3.81		
120.00	444.95	3.75		

a *X*: ln of percent of OTB remaining.

b Linear part regression equation.

c Correlation coefficient.

d Half-life.

e Intrinsic clearance.

## 4. Conclusions

A reliable and precise LC-MS/MS methodology was established for assaying the newly approved drug OTB in plasma. The established method is very sensitive (LOD = 1.12 ng mL^−1^), eco-friendly (using small volumes of organic solvents), rapid (run time = 3 min.) and accurate (*R*% = 98.4 ± 2.5%). The adopted LC-MS/MS methodology was utilized for OTB metabolic stability assessment in RLM matrix using two terms: *in vitro t*_1/2_ (2.71 min) and intrinsic clearance (48.8 mL min^−1^ kg^−1^). From these two values, we concluded that OTB can be classified among low-extraction ratio drugs and hence can be slowly eliminated from the human body.

## Authors' contribution

MWA, AAK and HWD participated in putting the design of the research in addition to guiding the practical work. HWD, ASA and MWA performed the optimization and method validation experiments and manuscript writing. All the authors participated in revising and approving the final draft of the manuscript.

## Conflicts of interest

The authors declare that they have no competing interests.

## List of abbreviations

CE: Collision energy
EGFR: Epidermal growth factor receptor
FV: Fragmentor voltage
HQC: High quality control
IS: Internal standard
IS normalized MF: Internal standard normalized matrix effect
LC-MS/MS: Liquid chromatography tandem mass spectrometry
LOD: Limit of detection
LQC: Lower quality control
LOQ: Limit of quantification
MQC: Medium quality control
NSCLC: Non-small cell lung cancer
OTB: Olmutinib
PNT: Ponatinib
QC: Quality control
TKI: Tyrosine kinase inhibitor

## Supplementary Material
